# Aerial root formation in Oaxacan maize (*Zea mays*) landraces persists into the adult phase and is minimally affected by soil nitrogen and ambient humidity

**DOI:** 10.3389/fpls.2025.1607733

**Published:** 2025-07-11

**Authors:** Rafael E. Venado, Jennifer Wilker, Valentina Infante, Caitlin McLimans, Fletcher Robbins, Courtney Phillips, Claudia Irene Calderón, Jason G. Wallace, Jean-Michel Ané

**Affiliations:** ^1^ Department of Bacteriology, University of Wisconsin-Madison, Madison, WI, United States; ^2^ Department of Plant and Agroecosystem Sciences, University of Wisconsin-Madison, Madison, WI, United States; ^3^ Center for Applied Genetic Technologies, University of Georgia, Athens, GA, United States; ^4^ Escuela de Biología, Universidad de San Carlos de Guatemala, Guatemala City, Guatemala; ^5^ Department of Crop and Soil Sciences, University of Georgia, Athens, GA, United States; ^6^ Institute of Plant Breeding, Genetics, and Genomics, University of Georgia, Athens, GA, United States

**Keywords:** maize, aerial roots, nitrogen, humidity, landraces

## Abstract

Maize (*Zea mays* L.) is the most widely produced crop in the world, and conventional production requires significant amounts of synthetic nitrogen fertilizer, which has negative economic and environmental consequences. Maize landraces from Oaxaca, Mexico, can acquire nitrogen from nitrogen-fixing bacteria that live in a mucilage secreted by aerial nodal roots. The development of these nodal roots is a characteristic traditionally associated with the juvenile vegetative stage of maize plants. However, mature Oaxacan landraces develop many more nodes with aerial roots than commercial maize varieties. Our study shows that Oaxacan landraces develop aerial roots during the juvenile and adult vegetative phases and even during early flowering under greenhouse and field conditions. Surprisingly, the development of these roots was only minimally affected by soil nitrogen and ambient humidity. These findings are an essential first step in developing maize varieties to reduce fertilizer needs in maize production across different environmental conditions.

## 1 Introduction

Plant roots support plant growth by fulfilling essential water and nutrient acquisition and anchorage functions. Roots release a significant amount of the plant photosynthates in the surrounding soil, allowing plants to shape their rhizosphere microbiome (Pantigoso et al., 2022; Santangeli et al., 2024). Roots are also the site of intimate associations with symbiotic microbes, such as nitrogen-fixing bacteria (diazotrophs) or mycorrhizal fungi, which can improve plant nutrition and stress tolerance. Roots predominantly arise from the root apical meristem (RAM); Embryonic roots in maize, including the seminal and primary roots, are formed during embryogenesis and contain RAM, although they are not derived from it (Atkinson et al., 2014; Tai et al., 2016). Postembryonic root development in maize, whether below or above ground, can proceed via continued RAM activity or *de novo* organogenesis from various cell types (Hochholdinger et al., 2004; Atkinson et al., 2014). While the shoot apical meristem (SAM) initiates above-ground structures such as leaves and tassels, it also forms nodal roots (Thompson et al., 2015). Specifically, brace roots emerge from internodes, and adventitious roots can form on shoot tissues in response to environmental cues or wounding (Bellini et al., 2014; Yu et al., 2015)Regardless of their origin, these meristems serve as reservoirs of stem cells, perpetually generating new cells to sustain growth and differentiation (Benfey and Scheres, 2000).

**Figure 1:**
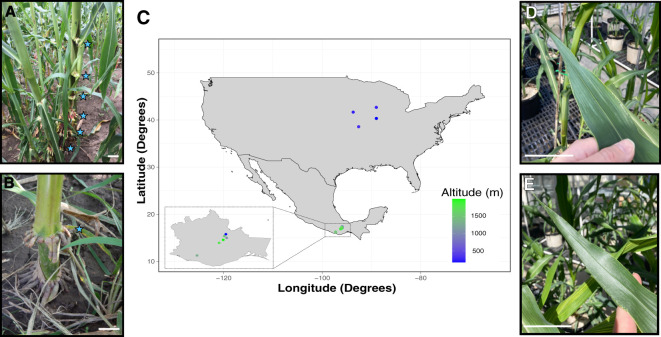
Maize landraces and exPVP lines and their phenotypes. **(A)** Maize landrace and its aerial roots. **(B)** exPVP PHP02 and its brace roots. Stars show the location of aerial or brace roots. **(C)** Geographical distribution (latitude, longitude, and altitude) of the maize genotypes used in this study. **(D)** Last leaf with epicuticular wax. **(E)** First leaf without epicuticular wax. Scale bar across all figures 5 cm.

Root systems differ vastly among angiosperm plants, and monocots exhibit a complex root structure distinct from eudicots. The monocot primary root undergoes decay and is rapidly substituted by a fibrous root system composed of adventitious nodal roots, also known as crown roots, originating at the stem base (Hochholdinger et al., 2004; Blizard and Sparks, 2020). Maize (*Zea mays* L.) features crown and above-ground nodal roots, often referred to as “brace roots,” that reach the ground and serve essential anchorage functions (Hostetler et al., 2021). However, some maize accessions also produce many nodal roots that never reach the ground (hereon referred to as “aerial roots”), and their function remained enigmatic for a long time (**Figure 1A**). In 2018, we reported that landraces from the Southern state of Oaxaca, Mexico develop many more aerial roots than other widespread maize varieties (**Figure 1B**) (Van Deynze et al., 2018). We also demonstrated that these maize landraces can acquire substantial amounts of nitrogen from diazotrophs that reside in a gel/mucilage produced by their aerial roots after rainfall (Van Deynze et al., 2018; Bennett et al., 2020; Pankievicz et al., 2022). Both brace and aerial roots originate from stem nodes derived from the SAM. This developmental process involves four stages: induction, where founder cells acquire the ability to divide; initiation, marked by the observable presence of root primordia in the node; emergence, during which the root primordium elongates; and growth, where elongation continues until the complete disappearance of the root cap (Itoh et al., 2005).

The root development process is intricate and dynamic, influenced by genetic and environmental factors. In maize, 21 distinct genes have been identified as affecting brace root development to varying degrees, as previously reviewed (Hostetler et al., 2021). Environmental factors, including water availability, temperature, microbes, and drought, often influence root development (Lynch et al., 2012). Some of these factors modulate plant hormones, significantly impacting root development. For instance, waterlogged conditions prompt the development of adventitious nodal roots, facilitated by the accumulation of auxin at the stem base. In contrast, drought stress alters abscisic acid (ABA) and auxin levels to sustain root elongation (Kazan, 2013). Plant hormones play a fundamental role in root development, and their interplay and accumulation affect different root types. Specific hormones, such as auxin and ethylene, positively influence nodal roots, while others, like cytokinin, act as antagonists, hindering their development, as reviewed previously (Singh et al., 2023). Nutrient levels also affect the root system. For example, maize exhibits increased lateral root growth under short-term low nitrogen levels, but prolonged deficiency hampers lateral root development (Gaudin et al., 2011; Sun et al., 2022).

Root development often correlates with other developmental transitions. In maize, brace and aerial root development have been traditionally associated with the juvenile vegetative stage of maize growth (Poethig, 1990; Hostetler et al., 2021). Above-ground nodal roots are sometimes observed at the adult stage but only after lodging (Sparks, 2023). However, Oaxacan landraces develop many more nodes with aerial roots than commercial maize accessions, and they do not exhibit lodging (Van Deynze et al., 2018). This observation suggests that these landraces may continue to form aerial roots after the juvenile stage or that the juvenile stage in these accessions is extended as in the *corngrass1* (*cg1*) mutant (Chuck et al., 2007). In maize, the transition from juvenile-adult vegetative phase shift is marked by the disappearance of leaf epicuticular wax, leaf hairs, cell wall composition, and insect and rust resistance changes. Leaves five or six possess epidermal cells responsible for wax production. However, a transition occurs beyond the sixth leaf, marked by differentiation in cell types, notably the development of bulliform cells and leaf hairs (Freeling and Lane, 1994; Moose and Sisco, 1994; Bongard-Pierce et al., 1996; Sylvester et al., 2001; Poethig, 2003; Riedeman et al., 2008; Riedeman and Tracy, 2010).

Here, we investigated whether Oaxacan landraces produce aerial roots during adult growth under greenhouse and field conditions. In field trials conducted in Wisconsin and Georgia, we investigated how environmental conditions affect the formation of aerial roots. Concurrently, we investigated the impact of environmental factors on various traits of aerial roots. Oaxacan landraces were cultivated under three nitrogen levels in one experiment, and in another experiment, they were subjected to differing relative humidity levels. Both experiments took place in greenhouse settings. It is crucial to comprehend the diverse factors contributing to the development of aerial roots if we aim to harness the untapped potential of these landraces in breeding initiatives to reduce nitrogen fertilizers in agriculture.

## 2 Materials and methods

### 2.1 Greenhouse study on aerial root development

In the winter of 2022, at the Walnut Street Greenhouse, Madison, Wisconsin (43.076222571138636, -89.42402327492367), two exPVP (PHZ51, PHP02), one heirloom (Hickory King), and three landraces (Oaxa233, Oaxa524, Oaxa733) were planted in a randomized design with six replications. Planting was done in the second week of February 2022. All landraces were obtained from the International Maize and Wheat Improvement Center (Spanish acronym CIMMYT), previously collected from southern Mexico, and the exPVP and giant heirloom were acquired from the American Germplasm Resources Information Network (GRIN). All material used in greenhouse and field experiments is listed in **Supplementary Tables 1**, **2**. Ten seeds per genotype were sown in individual segments of a 24-segment plastic tray filled with HP+ Promix potting media. At 14 days after seeding, 6 seedlings of similar size per genotype were each transplanted to seven-gallon pots (Classic 2800) filled with HP+ Promix potting media. Germination rate was similar across the genotypes except for PHP02 that was 50% rate. The greenhouse temperature conditions were established as 25°C during the day and 20°C at night, with a 12-hour light cycle maintained from 6 am to 6 pm. Plants were irrigated using an automated drip irrigation system three days per week, and they were watered manually once per week with 500 ml of fertilizer water (NPK: 20-10-20). Last leaf epicuticular wax production was used as a morphological marker to determine the transition between the juvenile and adult vegetative phases (Freeling and Lane, 1994; Bongard-Pierce et al., 1996). This trait was scored on each plant at the V10-V12 stage following the protocol described by Foerster (1913); days to anthesis were summed from the planting date to 50% anther dehiscence; the number of nodes with aerial roots longer than 1 centimeter was counted on each plant at anthesis (Foerster, 1913).

### 2.2 Wisconsin field studies on aerial root development

In the summer of 2021, at the West Madison Agricultural Research Station, Wisconsin (43.0610329663, -89.5325872441), three exPVP (PHZ51, PHP02, HB8229) and three landrace accessions (Oaxa139, Oaxa524, GRIN19897) were planted in single-row plots in a randomized complete block design with three biological replicates. Planting was done the first week of June. These landraces were selected from previous studies and field observations in nitrogen fixation in maize (Van Deynze et al., 2018). The exPVPs were chosen as materials adapted to the Midwest in the US (Mikel, 2006; Van Deynze et al., 2018). Stand counts were conducted in each plot to assess germination rate and synchrony, and no issues with germination were observed. The last leaf with epicuticular wax was scored on three plants per plot at the V10-V12 stage as previously described; days to anthesis were summed from planting date to anther dehiscence in 50% of tassels in a plot; the number of nodes with aerial roots was counted on three plants per plot at anthesis. Additionally, the number of nodes with aerial roots was recorded weekly on three plants per plot over nine weeks, from 63 DAP to 119 DAP. In the summer of 2022 at the Hancock Agricultural Research Station in central Wisconsin (44.11985775008647, -89.53536890800639), three exPVPs (PHZ51, PHP02, and HB8229) and four landraces (Oaxa139, Oaxa524, Oaxa733, and GRIN19897) were planted in single-row plots in a randomized complete block design with three plot biological replicates and per replicate tree independent plant were measured. The number of nodes with aerial roots was recorded at 71, 101, and 116 DAP. **Supplementary Table 2** provides access to information about our experiments.

### 2.3 Georgia field study on aerial root development

In the summer of 2022 at the University of Georgia’s Iron Horse Farm, Georgia (33.72707583516916, -83.30204915982816), 11 landrace accessions (Oaxa233, Oaxa229, Oaxa306, Oaxa310, Oaxa141, Oaxa139, Oaxa524, Oaxa612, GRIN19970, Oaxa622, Oaxa182) were planted and screened in single-row plots organized by genotype in a complete block design with three replicates. Planting was completed in the second week of May. Stand counts were conducted in each plot to assess germination rate and synchrony, and no issues with germination were observed. The phenotypes evaluated during the Wisconsin field study 2021 were also assessed, although the aerial root phenotypes were checked on five plants per plot. **Supplementary Table 2** provides access to information about our experiments.

### 2.4 Greenhouse study on soil nitrogen rates

Three maize accessions (GRIN19770, Oaxa139, and Oaxa229) were planted at the University of Wisconsin-Madison’s Walnut greenhouse (Madison, WI) under controlled conditions of 25°C during the day, 20°C at night, and a 12-hour light cycle from 6 am to 6 pm. Due to the light-sensitive nature of these maize accessions, all windows and walls were covered with black tarps. Seven-gallon pots (Classic 2800) were filled with a mixture of autoclaved calcined clay, also known as Turface(R) (Turface Athletics MVP), and sand in a 4:1 v/v ratio, adapted from Kottkamp et al. (Kottkamp et al., 2010). The high temperatures during calcination cause the clay to expand, creating a porous structure. This structure provides physical and chemical stability, allowing for aeration to plant roots while retaining water within its pore network. This substrate selection aimed to minimize variability in soil potting media, ensuring consistent growing conditions for the plants. Each pot was positioned over a 13-inch plastic pot saucer to prevent the loss of fertilization treatment through the pots’ drainage holes. These trays served as a means to ensure that all the plants had access to the same amount of nitrogen treatment. To investigate the effect of nitrogen fertilization on the number of nodes with aerial roots, three different nitrogen rates were tested: 175 ppm (high), 120 ppm (medium), and 70 ppm (low). These were achieved using various ratios of Hoagland’s solutions with and without nitrogen (HOP01 and HOP03 from Caisson Labs). The nitrogen in the HOP01 mix was provided by ammonium phosphate, calcium nitrate, and potassium nitrate, which together contributed 175 ppm of nitrogen. The HOP03 mix did not contain any source of nitrogen. To achieve the desired nitrogen level, a plastic container was filled with a mix of 70% HOP01 solution and 30% HOP03 solution. For the low nitrogen level, 40% of the HOP01 solution and 60% of the HOP03 solution were used. Each treatment comprised three replicates, resulting in a total of 27 pots that were randomly distributed across the greenhouse room. Synchronized germination was similar across the genotypes. Plants were watered five days a week, receiving 500 mL of the corresponding nitrogen treatment twice a week. The number of nodes with aerial roots was assessed thrice during the experiment at 80, 90, and 132 DAP. Aerial roots were counted as such only if they were not touching the ground and measured 1 cm or more long. In contrast, the root diameter of the aerial roots was measured once at 132 DAP, and three roots were selected randomly from the fourth node (or the highest node if no roots were present in the fourth node). **Supplementary Table 2** provides easy access to information about our experiments.

### 2.5 Greenhouse study on ambient humidity levels

The maize genotypes Oaxa524 and PHP02 were cultivated in two rooms at the Walnut Street Greenhouses at the University of Wisconsin-Madison. They were grown in seven-gallon pots (Classic 2800) filled with Pro-Mix LP15 medium, with daytime temperatures maintained at 28°C and nighttime temperatures at 25°C. Seeds were scored for germination with no deviation between genotypes. Pots were randomized in the greenhouse, and plants were exposed to a 12-hour light photoperiod from 6 am to 6 pm and 12 hours of darkness. A humidifier system (Smart Fog, Reno, Nevada) was employed to regulate humidity levels, ensuring a high humidity of 75% and a low humidity of 30%. Plants were irrigated twice a week with municipal water and received 500 mL of a 20-10–20 NPK fertilizer solution weekly. At the flowering stage, the plants were phenotyped for three parameters: the number of nodes with aerial roots, the number of roots on the top node, and aerial root diameter with a caliper. These measurements were carried out using the established procedure as previously described. **Supplementary Table 2** provides easy access to information about our experiments.

### 2.6 Data analysis

Statistical analysis and data plotting were done using R statistical software (version 4.2.1) (R Core Team and Others, 2013). For parametric data, ANOVA and pairwise comparisons with Tukey’s Honestly Significant Difference Tukey HSD were performed with the package “multcompView (version 0.1 - 8)” (Graves et al., 2024). For non-parametric data, a Wilcoxon test was performed with the package “ggpubr (version 0.6.0)” (Kassambara, 2023). Correlation analysis was performed with the package “correlation (version 0.8.6)” (Makowski et al., 2022). ANOVA assumptions were reviewed with the package “emmeans (version 1.11.1)” (Estimated Marginal Means, aka Least-Squares Means, n.d.). All plots were generated with the package “ggplot2 (version 3.5.0.9000)” (Wickham, 2009). Pairwise comparisons for all experiments are provided in **Supplementary Data 1**.

## 3 Results

### 3.1 Oaxacan maize landraces develop elongated aerial roots during the adult vegetative and reproductive phases in controlled greenhouse conditions

**Figure 2:**
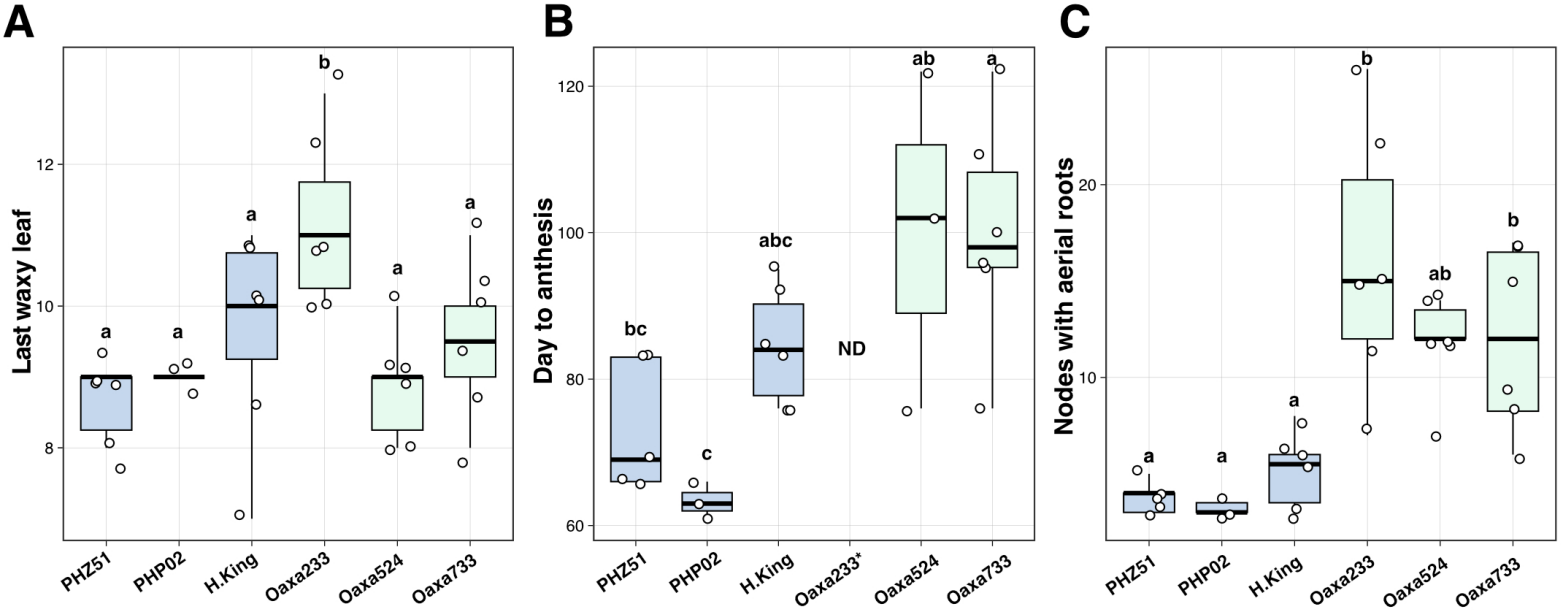
Comparison of exPVP, heirloom, and landrace genotypes in a greenhouse setting winter 2022. **(A)** Last leaf with epicuticular wax. **(B)** Days to anthesis. The landrace Oaxa233 did not flower, except in a single replicate, and was therefore excluded from the analysis (Not Determined, ND). **(C)** Number of nodes bearing aerial roots at anthesis. The number of biological replicates was between three to six. Landraces (green boxes) exhibit a longer time to anthesis and have more nodes with aerial roots while still showing a similar transition from juvenile to adult stage compared to the exPVP and Hickory King lines (blue boxes). ANOVA and Tukey's Honestly Significant Difference tests were performed with the package multcompView (version 0.1- 8).

**Figure 3:**
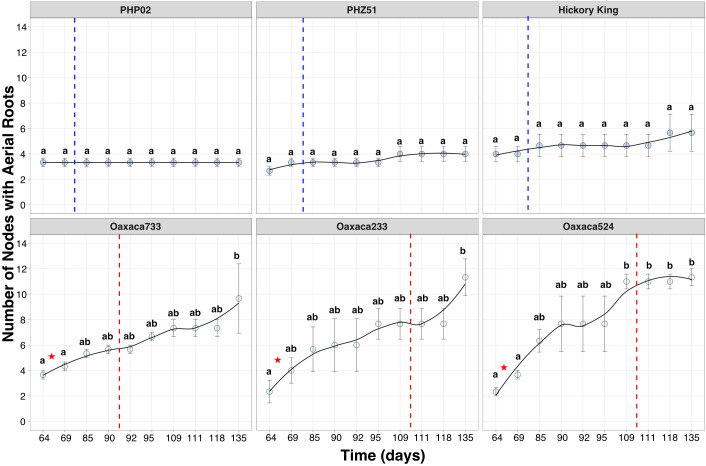
Quantification of aerial roots across six maize genotypes over time. Transitions from juvenile to reproductive and tasseling days are displayed. The scatter plot illustrates the number of nodes with aerial roots, showing that landraces have a significantly higher count than the exPVP and Hickory King varieties. Additionally, landraces continue to develop aerial roots for several days after tasseling. The blue dotted line indicates the tasseling period for conventional maize lines (70-85 days after planting), while the red dotted line marks the tasseling period for landraces (95-100 days after planting). The red star indicates the transition from juvenile to adult vegetative stage in the landraces (after 65-70 days). An ANOVA test was performed using the multcompView package (version 0.1-8). Tukey's Honestly Significant Difference was performed with the package "multcompView (version 0.1 - 8).

A greenhouse study was conducted to examine growth phases and aerial root formation in two exPVP inbreds (PHZ51, PHP02), one heirloom (Hickory King), and three Oaxacan landrace accessions (Oaxa233, Oaxa524, Oaxa733) under controlled conditions (**Figure 1C**). Leaf epicuticular wax was used as a marker for the juvenile-to-adult vegetative phase transition (**Figures 1D, E**). Growth of the last leaf with epicuticular wax was only significantly different for the landrace Oaxa233 compared with the other genotypes (**Figure 2A**; **Supplementary Data 1**). PHZ51, PHP02, and Oaxa524 transitioned to the adult phase after the growth of leaf nine, whereas Hickory King, Oaxa233, and Oaxa733, on average, transitioned one to two leaves later. The exPVP genotypes generally exhibited narrower variability for this trait. In contrast, the heirloom and landrace genotypes showed wider-ranging values, probably because the exPVP lines are genetically uniform inbreds, while the heirloom and landraces are heterogeneous and outbred. Anthesis (male flowering) marks the transition from the adult vegetative to the adult reproductive phase. Under greenhouse conditions with a 12-hour photoperiod, days to anthesis varied significantly, with the exPVPs and Hickory King tasseling earlier than the landrace genotypes Oaxa524 and Oaxa733 (**Figure 2B**). PHZ51 and PHP02 reached anthesis in less than 70 days, Hickory King in an average of 85 days, and the landraces in over 95 days post-planting. Oaxa233 appears to be photoperiod sensitive, and only one replicate produced a mature tassel thus was removed from the analysis. At anthesis, the number of nodes with aerial roots was quantified. The exPVP and heirloom genotypes produced significantly fewer nodes with aerial roots than landrace accessions (**Figure 2C**). For example, PHZ51 produced significantly fewer nodes with aerial roots than Oaxa524 (p = 0.029) and Oaxa233 (p = 0.0004). In addition, the adult vegetative phase (the time between forming the last leaf with epicuticular wax and anthesis) was longer in the landrace accessions than in the exPVP and heirloom genotypes (**Supplementary Figure 1**). Potential correlations were investigated among the number of nodes with aerial roots, days to anthesis, and the last waxy leaves. Our analysis revealed positive correlations among all traits in landraces and exPVP (**Supplementary Figure 2A**). Specifically, we observed a moderately positive correlation between the number of days to anthesis and the number of nodes with aerial roots (r = 0.66). In contrast, the correlations between the other pairs were very low (**Supplementary Figure 2B**). The number of nodes with aerial roots was also assessed at various intervals between 64 and 135 days after planting (DAP) (**Figure 3**). PHP02 and PHZ51 grew no additional nodes after tasseling (average 70 DAP) and averaged three nodes with aerial roots in both exPVPs (**Figure 3**; **Supplementary Figure 1**). In contrast, the larger Hickory King variety flowered at an average of 85 DAP and averaged five nodes with aerial roots per plant. Intriguingly, the landraces continued producing aerial roots even after anthesis, typically after 100 DAP (**Figure 3**; **Supplementary Figure 1**). These findings suggest some Oaxacan landraces (Oaxa524 and 733) continue developing aerial roots into the adult reproductive stage, unlike the other maize lines tested here.

### 3.2 Oaxacan maize landraces exhibit significant aerial roots during the adult vegetative and reproductive stages in Wisconsin and Georgia under field conditions

#### 3.2.1 2021 field experiment in Wisconsin

To study growth-stage traits of landrace and exPVP maize in the field, a trial was conducted in the summer of 2021 at the West Madison Agricultural Research Station, Wisconsin (WI). Three exPVP varieties (PHZ51, PHP02, HB8229) and three landrace accessions (Oaxa139, Oaxa524, GRIN19897) were evaluated. Significant differences were found for the last waxy leaf between genotypes (**Supplementary Figure 3A**; **Supplementary Data 1**); however, similar to the greenhouse waxy leaf results (**Figure 2A**), there were no clear patterns differentiating the exPVP genotypes and landrace accessions. For example, the last waxy leaf for PHZ51 did not differ significantly from that of Oaxa139 or Oaxa524, nor did the last waxy leaf for HB8229 differ significantly from PHP02 or GRIN19897. Interestingly, the last waxy leaf findings appear consistent between PHZ51 and Oaxa524, both in greenhouse and field conditions. Days to anthesis were measured to ascertain the length of the vegetative stage under field conditions. Significant differences were observed, with genotypes PHP02 and Oaxa139 flowering in under 60 days, while the landraces Oaxa524 and GRIN19897 flowered around 100 DAP (**Supplementary Figure 3B**; **Supplementary Data 1**). Three distinct categories of days to anthesis were apparent. An early-flowering group (PHP02 and Oaxa 139) reached anthesis in less than 60 DAP. The intermediate-flowering group (PHZ51 and HB8229) reached anthesis in under 70 DAP. Finally, the late-flowering group (Oaxa524 and GRIN19897) had a significantly longer adult vegetative phase, reaching anthesis more than one month later, at approximately 100 days after pollination (DAP). The number of nodes with aerial roots, those regions on the stem generating roots without touching the ground, was counted at anthesis. Significant differences were observed among the genotypes (**Supplementary Figure 3C**; **Supplementary Data 1**). The exPVPs had aerial roots on one to two nodes, whereas landraces had roots on two to six nodes. The landrace accessions Oaxa524 and GRIN19897, which formed aerial roots on significantly more nodes, were the same accessions that took substantially longer to reach the adult reproductive stage (**Supplementary Figures 3B, C**; **Figure 3**). In the field, we also recorded the number of nodes bearing aerial roots weekly from nine weeks (63 days) to 17 weeks (119 days) post-planting (**Supplementary Figure 4**). This period encompassed the adult reproductive phase of the trial, starting from when PHP02 (the earliest flowering genotype) reached anthesis and continuing until most plants had reached physiological maturity. The exPVP lines (PHZ51, PHP02, and HB8229) all ceased forming aerial roots on nodes once they reached reproductive maturity (anthesis), whereas GRIN19897, Oaxa 524, and Oaxa139 formed an additional node with aerial roots post-anthesis. One particularly close comparison is PHP02 and Oaxa139, which flowered at a similar time (58 and 59 DAP, respectively). PHP02 had two nodes with aerial roots at anthesis and did not develop more nodes with aerial roots, whereas Oaxa139 grew an additional node with aerial roots after anthesis. Landrace genotypes GRIN19897 and Oaxa524 had a long adult vegetative phase and developed five to six nodes with aerial roots by anthesis, and Oaxa524 developed one more node after anthesis (an average of seven nodes).

#### 3.2.2 2022 field experiment in Georgia

A field trial was established at the University of Georgia Iron Horse Plant Sciences Farm (GA; summer 2022) to observe growth-stage-associated traits in a completely different environment. In this study, six landrace accessions, including two from previous WI studies (Oaxa139, Oaxa524) and four other landrace accessions (Oaxa141, Oaxa306, Oaxa612, and Oaxa622), were grown (**Supplementary Figure 5**). In GA, the final waxy leaf appeared with a median range between leaf 7 and leaf 10 (**Supplementary Figure 5A**), while in WI, it ranged from leaf 9 to leaf 10 (**Supplementary Figure 3A**). Oaxa139 and Oaxa524 exhibited an earlier waxy leaf transition in GA, reaching the final waxy leaf at leaf 7, whereas in WI, the final waxy leaf was at leaf 9. In WI, the anthesis range among landraces spanned 40 days, largely due to Oaxa139. In GA, this range extended to 50 days because Oaxa306 reached anthesis significantly earlier, at 52 DAPs (**Supplementary Figure 5B**). Interestingly, Oaxa139 reached anthesis in less than 60 days in WI, yet it took over 90 days to reach anthesis in GA (**Supplementary Figures 3B**, **5B**). Post-anthesis also significantly differed between the landraces and the exPVPs in different environments (**Figure 2**; **Supplementary Figures 3**, **5**). The number of nodes with aerial roots at tasseling differed among landrace accessions grown in GA and ranged from 1 to 14 nodes (**Supplementary Figure 5C**). Oaxa139 and Oaxa524 formed aerial roots on more nodes in GA than in WI.

#### 3.2.3 2022 field experiment in Wisconsin

In 2022, a trial was conducted to assess trait expression for a second year, using the same exPVPs and landraces as the previous Wisconsin greenhouse experiment, along with one additional accession. Due to the field’s distance (approximately 2 hours from campus), aerial root nodes were recorded at three key points: 71 DAP (before most accessions flowered), 101 DAP, and 116 DAP (after most accessions flowered) (**Supplementary Figure 6**). Consistent with earlier findings, Oaxa524 exhibited the highest average number of nodes with aerial roots (more than 7). These results confirm that maize landraces (e.g., Oaxa524) consistently produce nodes with aerial roots, regardless of the environmental context.

### 3.3 Soil nitrogen rates have a limited effect on maize aerial root development

**Figure 4:**
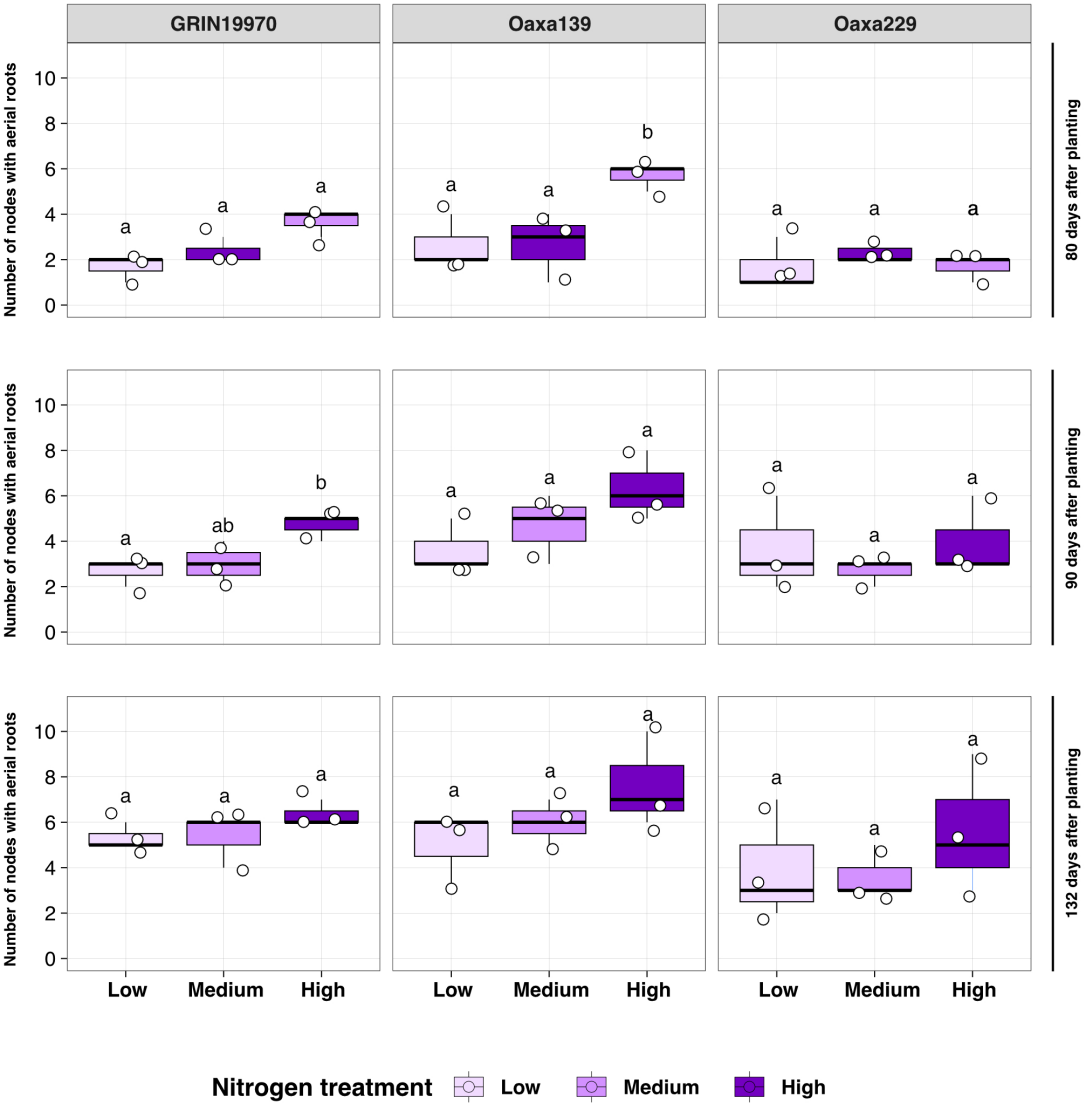
Aerial root development under different nitrogen treatments in maize landraces. The number of nodes with aerial roots was quantified in three landraces (GRIN19770, Oaxa139, and Oaxa229) under three different nitrogen treatments: low (70.36 nitrogen ppm), medium (123.14 nitrogen ppm) and high (175.91 nitrogen ppm). Overall, no significant differences were observed at different times in the landraces. ANOVA and Tukey's Honestly Significant Difference were performed using the package multcompView (version 0.1- 8) with R (version 4.2.1).

Insufficient nitrogen hinders plant growth and affects root architecture traits (Stitt and Krapp, 1999; Anas et al., 2020). A greenhouse experiment tested how nitrogen levels impact aerial root growth in three Oaxacan landraces. Nitrogen was applied at 70, 120, and 170 ppm. The number of nodes with aerial roots was recorded at 80, 90, and 132 DAP (**Figure 4**). No significant differences were observed when comparing the means of each nitrogen treatment within each accession and date, except for accession Oaxa 139, which showed a significant difference between high nitrogen and the other two treatments at 80 DAP. Nitrogen levels did not affect traits such as aerial root diameter, stalk diameter, and plant height (**Supplementary Figure 7**). These results indicate that varying levels of available soil nitrogen do not significantly impact the development of traits related to aerial root development in Oaxacan maize landraces.

### 3.4 Ambient humidity impacts minimally maize aerial root development

**Figure 5:**
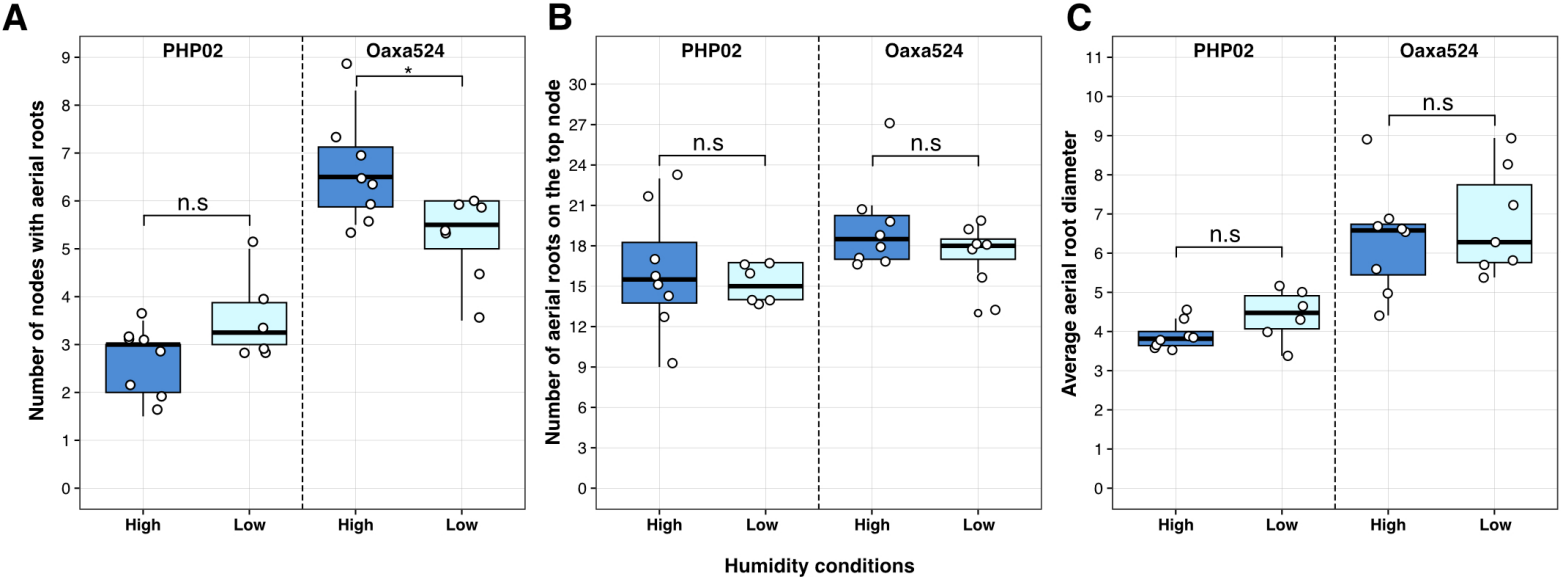
Effect of humidity on aerial root development in an exPVP (PHP02) and Oaxacan landrace (Oaxa524) accession. Different phenotypes were measured in the maize accessions after flowering undeer a high (75%) and low (30%) of relative humidity. **(A)** number of nodes with aerial roots. **(B)** Number of roots on the top node. **(C)** Average aerial root diameter at the top node. Only the number of nodes with aerial roots was affected in Oaxa 524. A Wilcoxon test was performed in R (version 4.2.1) with the package ggpubr (version 0.6.0). Significant levels *p-value < 0.05 and n.s. not significant.

The Oaxacan landraces grow in a high-humidity environment, which enables mucilage secretion from their aerial roots (Van Deynze et al., 2018; Bennett et al., 2020; Pankievicz et al., 2022). Our study aimed to investigate the effect of humidity on the development of aerial roots in the Oaxacan landrace Oaxa524, which is known for producing numerous nodes with aerial roots under various field and greenhouse conditions. We also used the exPVP PHP02 as a control. We tested two different humidity levels, 30% and 75%. Our experiment focused on measuring the number of nodes with aerial roots, their diameter, and the number of aerial roots at the top node. These parameters are associated with mucilage production (Pankievicz et al., 2022). In the Oaxa524 landrace, there was a slight increase of one node with aerial roots under high humidity (p = 0.021). However, this effect was not observed in PHP02 (**Figure 5A**). Additionally, no significant differences were observed in root diameter or the number of roots at the top node under varying humidity conditions (**Figures 5B, C**). These findings indicate a positive but limited effect of humidity in promoting the development of aerial root nodes in this Oaxacan maize landrace.

## 4 Discussion

### 4.1 Above-ground nodal root development is not a reliable marker for the juvenile stage in tropical maize landraces

The development of above-ground nodal roots (brace and aerial roots) in maize has traditionally been associated with the juvenile stage (Poethig, 1990; Hostetler et al., 2021). Mutants such as *Corngrass1* (*cg1*) are well known to develop five to six nodes with aerial roots due to an extended juvenile phase, as delayed flowering occurs 1 or 2 weeks, depending on the inbred background (Poethig, 1988, 2003; Chuck et al., 2007). Numerous maize accessions can develop such roots during the adult stage in response to lodging (Sparks, 2023). However, we showed here that the increased number of nodes with aerial roots in Oaxacan accessions is not due to an extended juvenile phase but to the production of these nodal roots at the adult stage without lodging. Apart from inherent diversity, numerous genes influence nodal root growth, acting as negative regulators (Hostetler et al., 2021). For example, knocking down the *teosinte glume architecture1 (tga1)* gene resulted in an increased number of nodal roots *(* Wang et al., 2015 *)*. Intriguingly, many of these genes are interconnected with flowering and responses to gibberellic acid, as reviewed in (Hostetler et al., 2021). Nonetheless, flowering in maize is a complex trait governed by many loci with modest impacts (Buckler et al., 2009). This trait is a pivotal adaptation linked to geographical positioning (Ziello et al., 2009; Crimmins et al., 2010). Our observations reveal that the landraces tested here (all cultivated above 1,500 meters) exhibit delayed/late flowering compared to the exPVP lines. Understanding the genetic factors underlying these two traits requires a thorough investigation. Despite research indicating no connection between flowering time and brace root development, it is imperative to investigate the potential presence of genetically correlated variables in these landraces (Reneau et al., 2020). Tropical genotypes are characterized by late flowering, significant height, and increased biomass (leaves) when grown in temperate latitudes (Warrington and Kanemasu, 1983). Wisconsin and Georgia experience distinct photoperiods, with Wisconsin receiving up to 15 hours of daylight at its peak, in contrast to Georgia’s 14 hours. This one-hour difference in daylight may account for why landraces tend to flower earlier in Georgia than in Wisconsin, reflecting the tropical origins of this material. Hence, delving into these variables is pertinent to gain deeper insights into their suitability for breeding initiatives. We evaluated the epicuticular wax as a morphological marker to determine the transition between juvenile and adult stages. In maize, juvenile leaves have epicuticular wax layers, whereas adult leaves lose this waxy layer and have crenulated epidermal cells (Sylvester et al., 2001). The environment affected this trait, as waxy leaves in Oaxa139 and Oaxa524 genotypes ceased to occur at an earlier growth stage in GA than in WI (**Figure 2**; **Supplementary Figures 3**, **5**). In our investigation, through scrutinizing the number of nodes with roots and various traits across three distinct environments, we observed that the landrace Oaxa524 exhibited higher performance compared to other landraces, particularly in terms of number of nodes with aerial roots (**Figures 2**, **3**; **Supplementary Figures 4**-**6**). This insight holds promise for breeding programs that incorporate aerial root traits into varieties suited for temperate climates, while introducing nitrogen fixation capabilities to reduce reliance on fertilizers. We recognize the need for further studies to determine the adaptability of these root traits across various environments, ranging from temperate to tropical regions. The selected exPVP inbred lines served as references, due to their extensive phenotypic and genotypic characterization and adaptation to temperate environments. However, we acknowledge the limitations of this comparison, particularly regarding maize diversity, as landraces are a source of unique alleles. In the future, generating hybrids between these exPVP lines and landraces will offer a more robust framework for assessing traits under local environmental conditions and better capturing heterotic effects. Additionally, the inclusion of tropical inbred lines of Mexican origin, such as the CIMMYT Maize Lines (CMLs), which were developed using landraces from Mexico. (Warburton et al., 2008) could significantly improve these comparisons. The CMLs are genetically closer to Oaxacan landraces and exhibit root phenotypes that are more similar to those found in the Oaxacan landraces. Future studies should incorporate both exPVP-derived hybrids and a broader panel of tropical lines, including CMLs, to enhance the generality of our findings and more accurately contextualize the unique traits of Oaxacan maize. Moreover, it has been noted that the aerial root development trait is not exclusive to Oaxacan landraces but is instead distributed across other regions of Mexico (Connolly et al., 2025). This expanded framework will also help clarify whether the observed phenotypic differences are primarily due to environmental adaptation or inherent genetic divergence across breeding histories. Currently, our analysis indicates that above-ground nodal root development should not be used as a marker for the juvenile stage in maize, at least among Oaxacan-like materials.

### 4.2 Soil nitrogen has a limited role in regulating maize aerial root development

Given the well-documented impact of nitrogen limitation on various physiological processes, many farmers worldwide rely on synthetic fertilizers to boost yields. However, their excessive use poses environmental risks, including water contamination through runoff and economic challenges, especially for small-scale farmers, who often experience the direct effects of price fluctuations influenced by global events (Arndt et al., 2023). With the growing interest in reducing synthetic nitrogen fertilizer use by enhancing nitrogen delivery through biological nitrogen fixation (BNF), it is crucial to understand whether an optimal nutritional rate supports the proper development of aerial roots, thereby maximizing the benefits of BNF. Our greenhouse experiment did not reveal any significant effect of soil nitrogen on aerial root development (**Figure 4**). In the future, it would be interesting to assess a broader range of nitrogen levels, encompassing both lower and higher concentrations than those used in this study. Additionally, the greenhouse experiment used Turface^®^ as the substrate, which, while advantageous for controlling nitrogen levels, differs considerably from field soil. Therefore, conducting similar experiments under field conditions in the future would be particularly interesting.

### 4.3 Ambient humidity exerts a limited influence on maize aerial root development

Plants display a variety of responses to changes in humidity levels. The biomass of aerial roots in indoor plants is increased due to the high relative humidity within households, facilitated by the indoor environment and misting systems, similar to those in our study (Sheeran and Rasmussen, 2023). Humidity affects the concentration of phytohormones. For instance, cucumbers’ gibberellic acid and auxin levels decrease in response to high humidity (Amin et al., 2021). Similarly, the ability to produce more aerial roots was found to be strongly affected by high relative humidity in tomatoes, resulting in a slight reduction in abscisic acid (ABA) levels and an increase in ethylene (Arve and Torre, 2015). Notably, applying exogenous auxin to stem cuttings increased adventitious roots in tomatoes, underscoring the significant influence of hormones and how various stimuli can further enhance this response (Guan et al., 2019). In maize, adventitious roots emerge from aerial nodes, and their development, elongation, and quantity are influenced by a complex interplay of genes and hormones (Singh et al., 2023). Elevated humidity slightly enhanced the number of nodes bearing aerial roots in the landrace Oaxa524 (**Figure 5A**). A phloem-based auxin response may contribute to this increased root formation in maize, akin to what has been observed in rice under saturated humidity (Chhun et al., 2007). Furthermore, ethylene, a plant hormone that accumulates during flooding and induces adventitious nodal roots, could be pivotal in developing nodes with aerial roots in maize. 1-Aminocyclopropane 1-carboxylic acid, which serves as an ethylene precursor, stimulates the generation of adventitious nodal roots in maize (Singh et al., 2023). Consequently, hormone precursors or their inhibitors emerge as a valuable approach to assess the progression of aerial root development in cereal crops. However, humidity did not impact the diameter or the number of roots in our maize accessions (**Figures 5B, C**). In contrast, a study of above-ground roots within the Araceae family showed that the root diameter increases under high humidity conditions (Sheeran and Rasmussen, 2023). Our results suggest that signals such as phytohormones may govern aerial root elongation in Oaxacan maize landraces at the adult stage without any lodging stimulation. Moreover, our study indicated that the impact of soil nitrogen and ambient humidity on aerial root development is limited, which may facilitate the use of this trait across diverse environments. However, as our experiments were conducted in a greenhouse, field studies are needed to confirm these findings.

### 4.4 Implications for biological nitrogen fixation in maize

In this study, our main objective was to explore how specific environmental factors influence the development of aerial roots in Oaxacan maize landraces and compare them with some exPVP adapted material to the Midwest. We recognize that these variables were analyzed in isolation, underscoring the importance of integrating them in the future to replicate field conditions more accurately. Much of the research was conducted in controlled greenhouse settings, limiting the impact of other environmental factors on aerial root development. Further investigation under diverse field conditions will be necessary to validate these findings. Our long-term goal is to enhance maize’s ability to acquire nitrogen through BNF, and thereby reduce its reliance on exogenous fertilizers. The finding that Oaxacan landraces continue to produce aerial roots into the adult stage has important consequences for breeding since this trait could be introgressed into elite varieties without the potential negative impacts of an extended juvenile phase, such as delayed flowering. The minimal but statistically significant GxE interaction we observed among field sites implies some environmental component to this trait, which may make some environments (e.g., arid ones) unsuitable for trait expression or at least suboptimal. Further research on aerial root production may help identify environment-independent alleles that could be used for breeding. Identifying the specific genes involved would enable more targeted allele mining or mutagenesis to generate the desired traits. Considering the extensive scale of global maize production, even a small reduction in nitrogen requirements could have significant economic and environmental impacts worldwide. This study set the foundation for incorporating these landraces into our breeding program, aiming to develop crop varieties that require less synthetic nitrogen fertilizer and contribute to the long-term goal of sustainable agriculture. This concept has been explored by other authors, who have studied different sets of maize landraces from various regions of Mexico and proposed screening across the Americas for these genetic resources (Connolly et al., 2025). In the future, we aim to share insights with the community on the agronomic performance and yield components of these landraces.

## Data Availability

The datasets presented in this study can be found in online repositories (https://doi.org/10.6084/m9.figshare.28727228.v1). The names of the repository/repositories and accession number(s) can be found in the article/**Supplementary Material**.
