# Supplementary figures and images for: Aerial root formation in Oaxacan maize (Zea mays) landraces persists into the adult phase and is minimally affected by soil nitrogen and ambient humidity
Source: Front Plant Sci. 2025 Jul 11;16:1607733. doi: 10.3389/fpls.2025.1607733 (PMC12289584; doi:10.3389/fpls.2025.1607733)

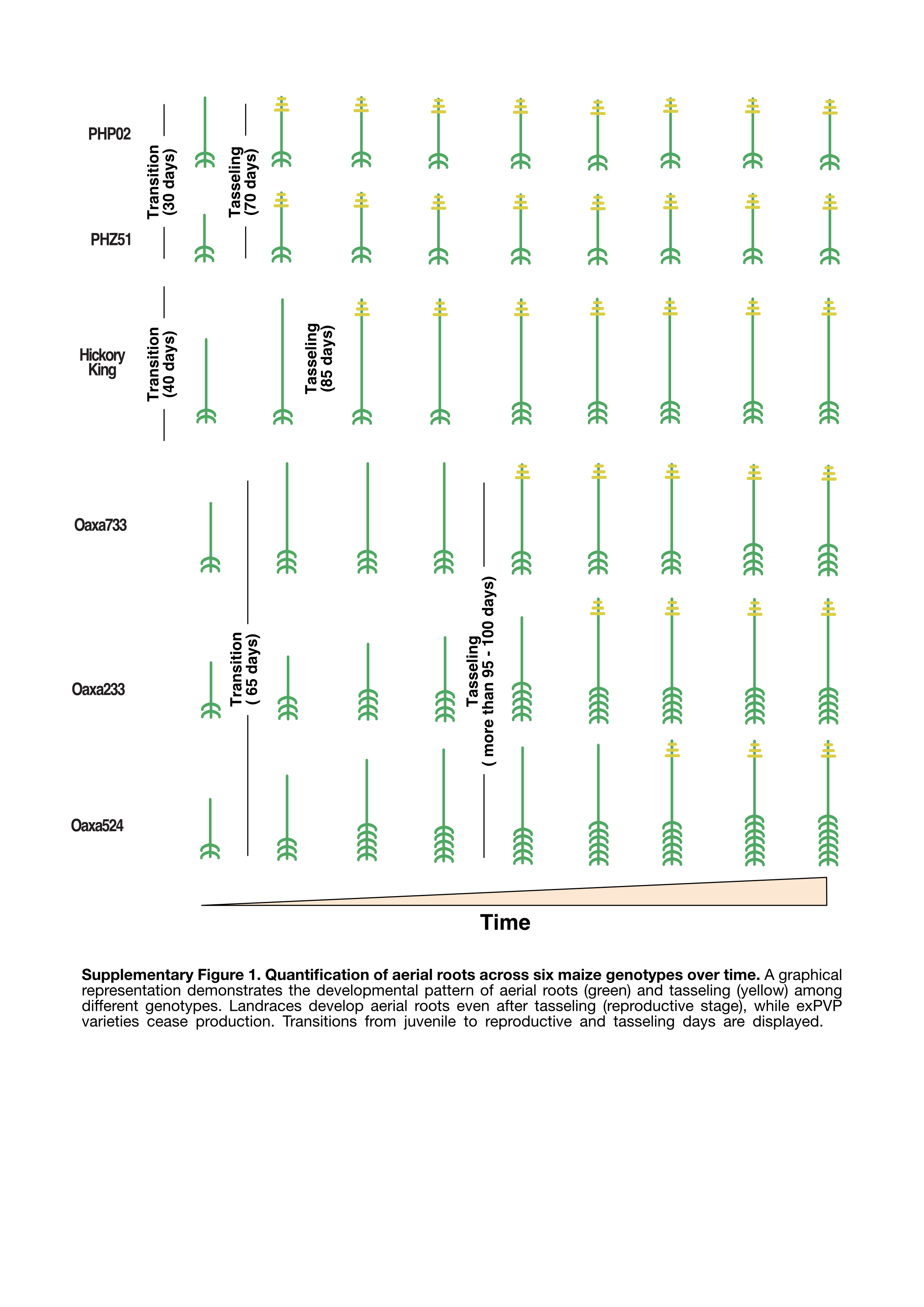

Supplement: Supplementary file 1 [file Image1.jpeg]

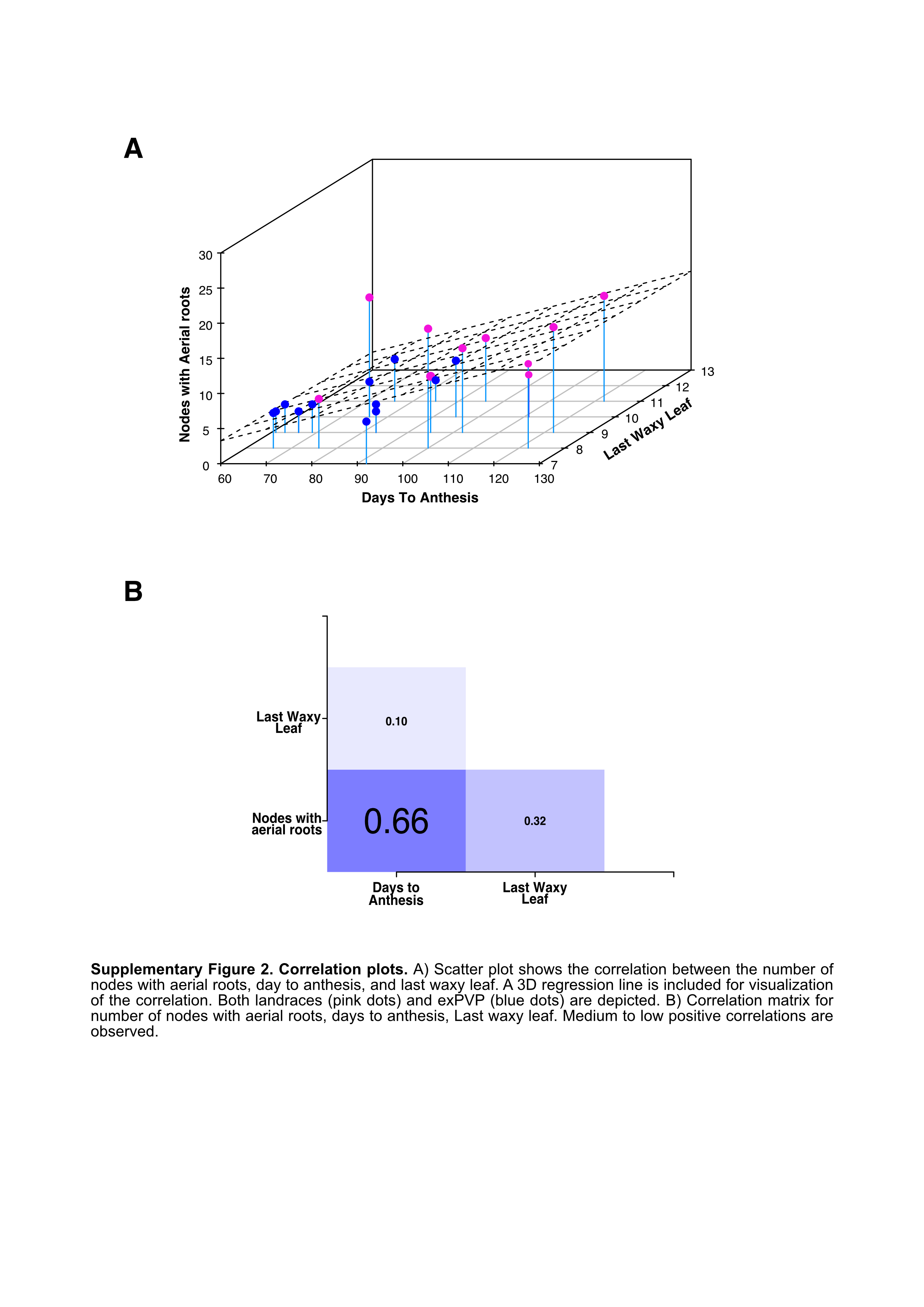

Supplement: Supplementary file 2 [file Image2.jpeg]

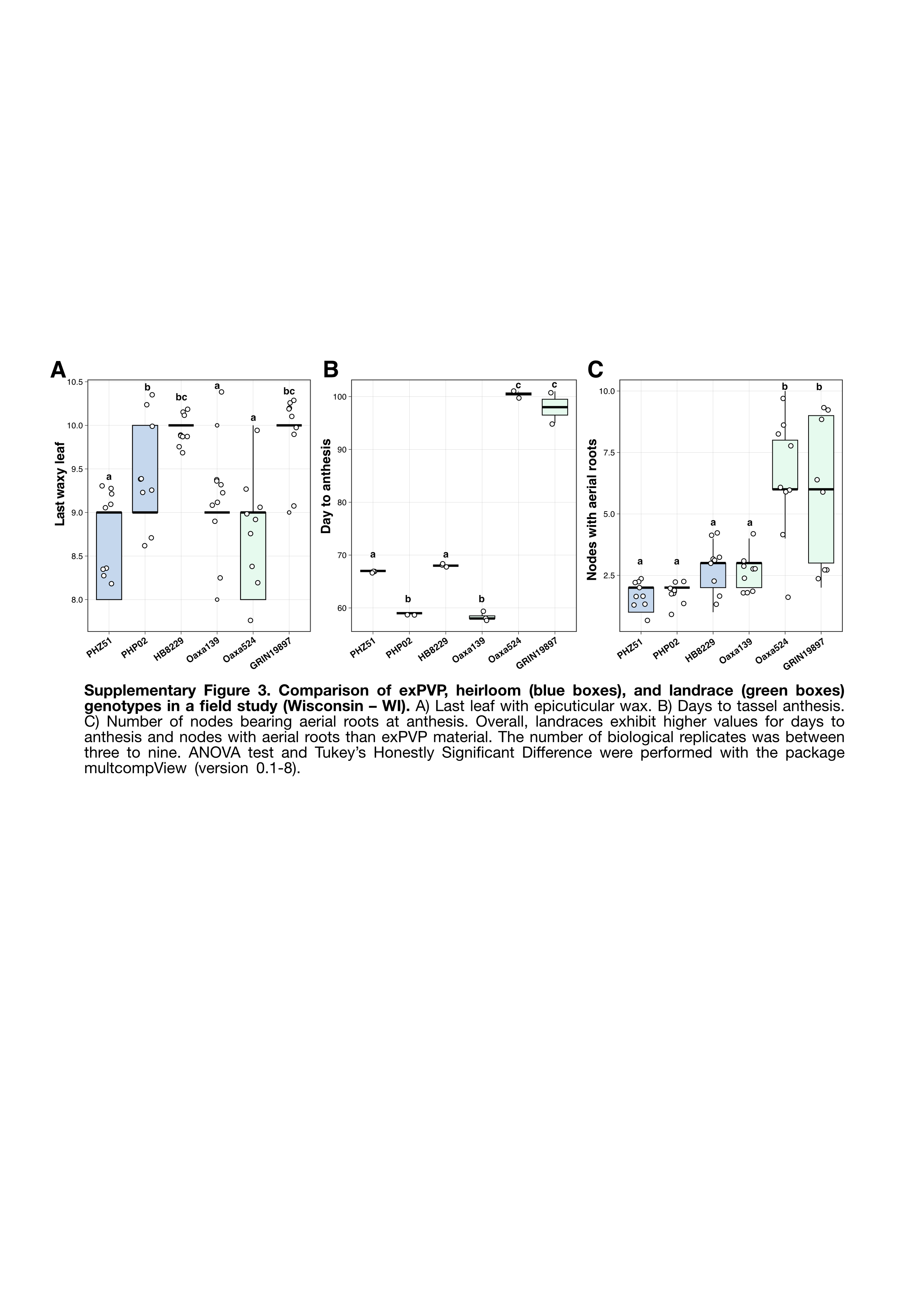

Supplement: Supplementary file 3 [file Image3.jpeg]

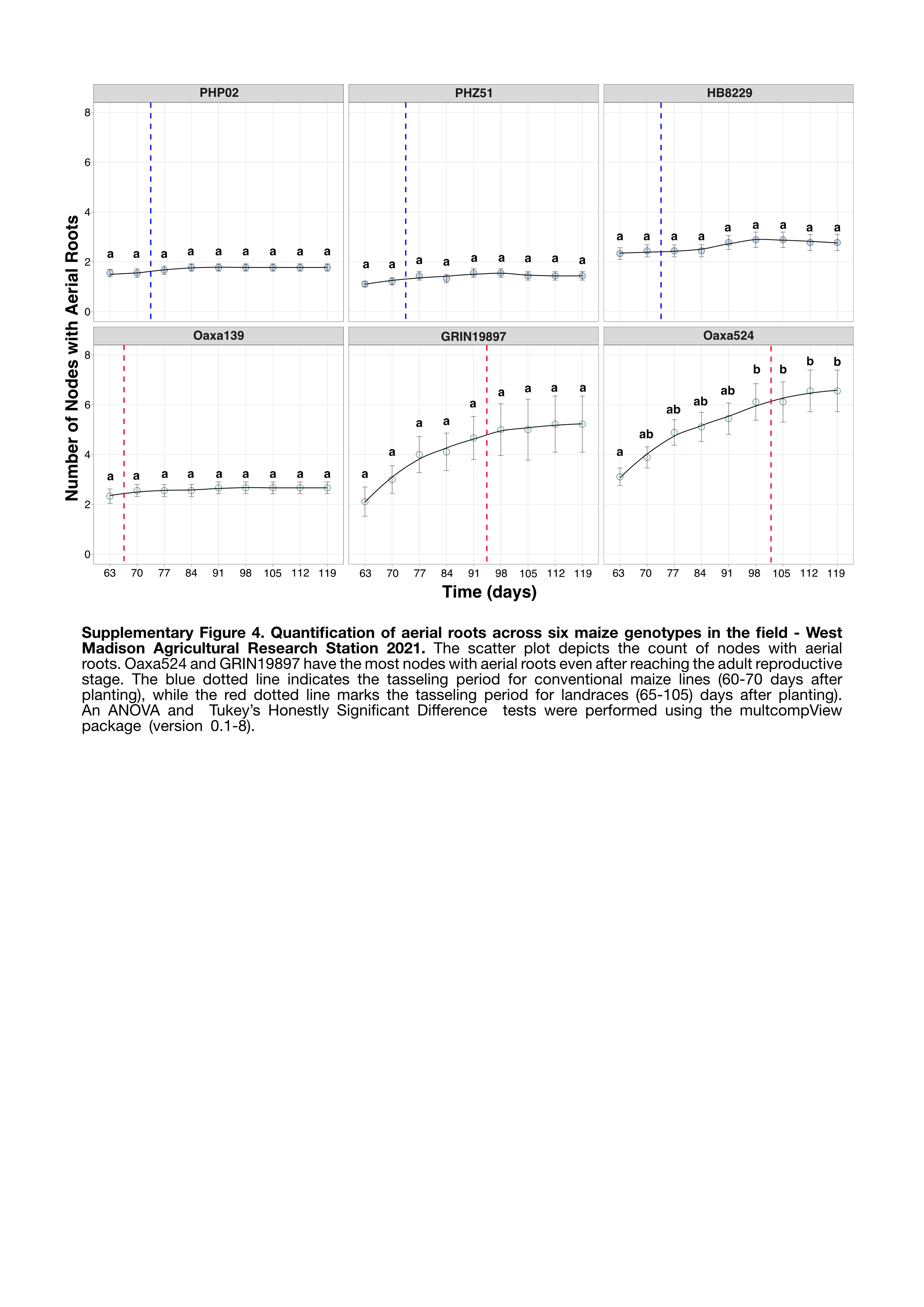

Supplement: Supplementary file 4 [file Image4.jpeg]

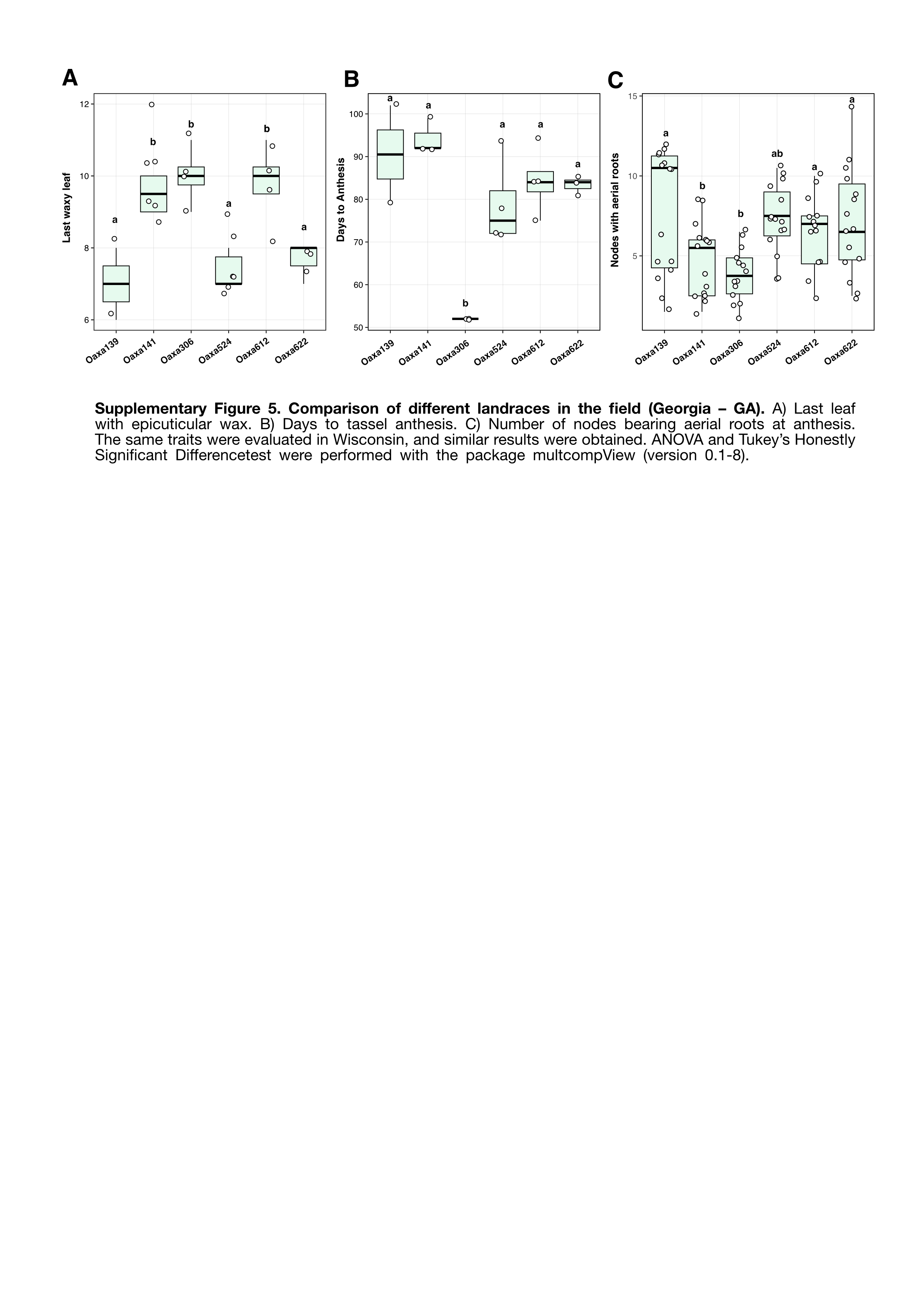

Supplement: Supplementary file 5 [file Image5.jpeg]

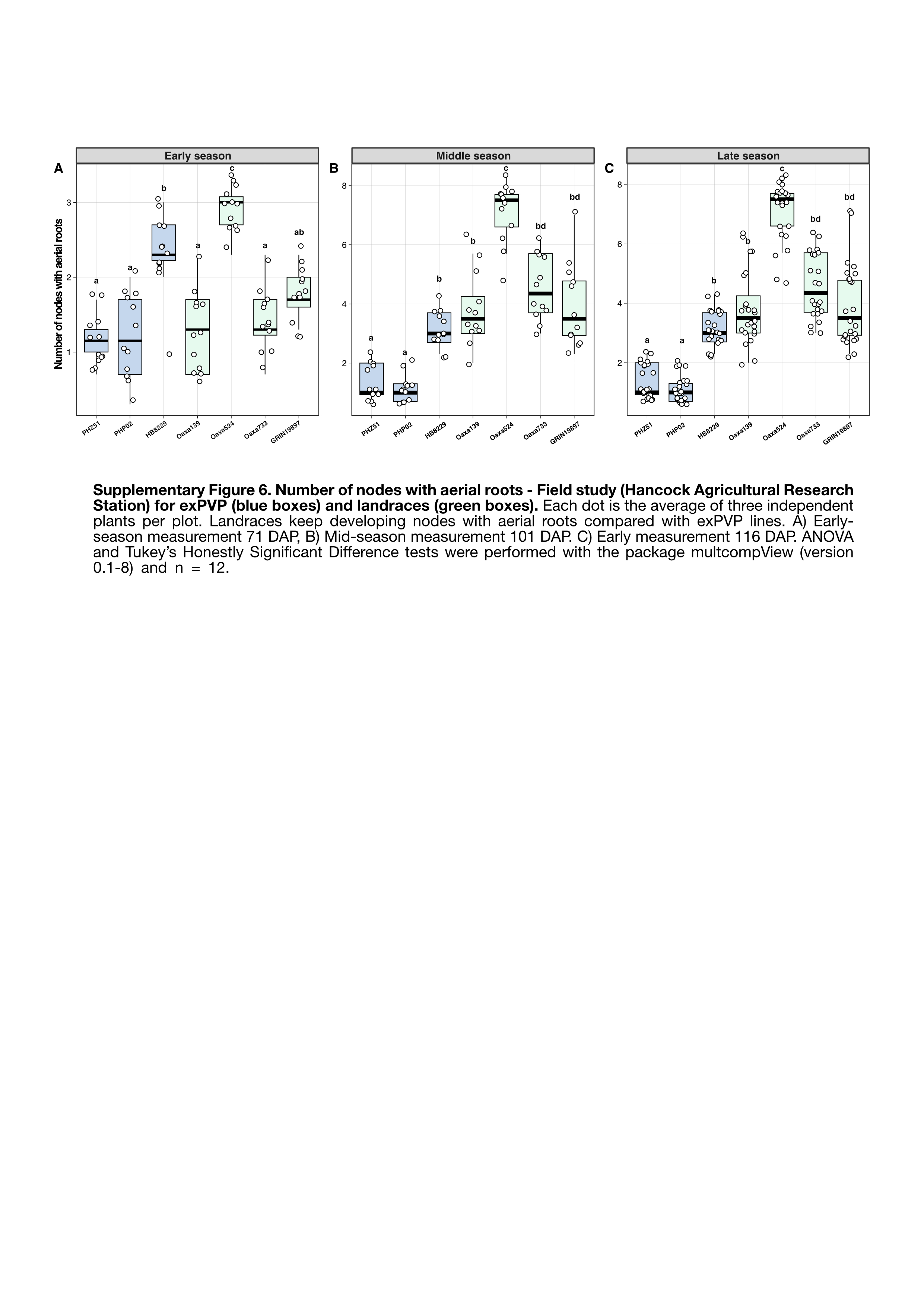

Supplement: Supplementary file 6 [file Image6.jpeg]

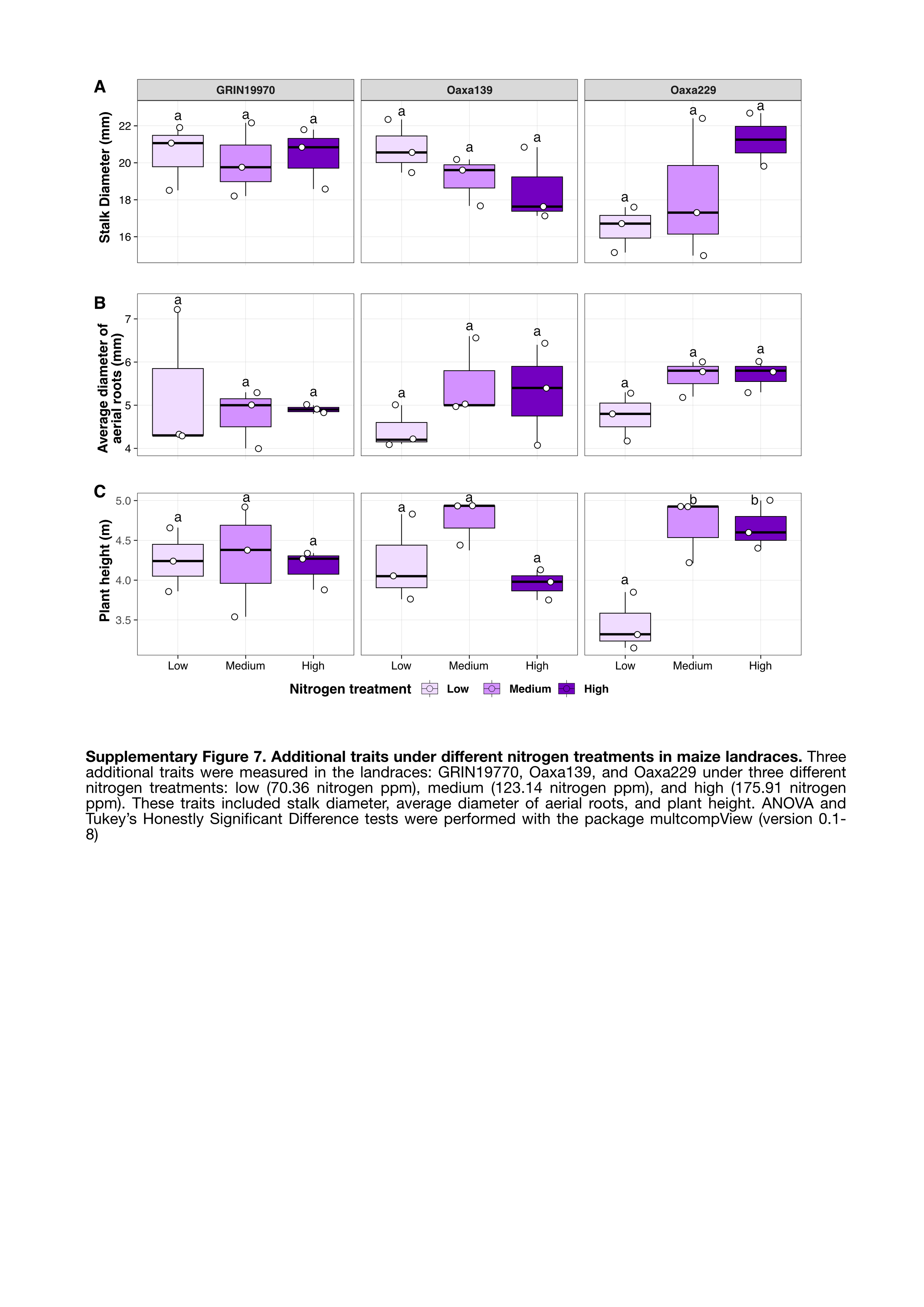

Supplement: Supplementary file 7 [file Image7.jpeg]
