# Supplementary material for: Aerial root formation in Oaxacan maize (Zea mays) landraces persists into the adult phase and is minimally affected by soil nitrogen and ambient humidity
Source: Front Plant Sci. 2025 Jul 11;16:1607733. doi: 10.3389/fpls.2025.1607733 (PMC12289584; doi:10.3389/fpls.2025.1607733)
Supplement: Supplementary file 8 [file Table1.docx]

**Supplementary Table 1**. Maize accessions (landraces or exPVP) used in this study.

| **Maize accession** | **Altitude (masl)** | **Latitude (degrees)** | **Longitude (degrees)** | **Source** |
| --- | --- | --- | --- | --- |
| OAXA139 | 1384 | 17.158801 | -95.913165 | CIMMYT |
| OAXA141 | 1384 | 17.158801 | -95.913165 | CIMMYT |
| OAXA229 | 1512 | 16.233634 | -97.28339 | CIMMYT |
| OAXA233 | 1512 | 16.233634 | -97.28339 | CIMMYT |
| OAXA306 | 1987 | 17.054241 | -96.072619 | CIMMYT |
| OAXA524 | 723 | 17.35 | -95.95 | CIMMYT |
| OAXA612 | 1651 | 17.253353 | -96.021444 | CIMMYT |
| OAXA622 | 723 | 17.35 | -95.95 | CIMMYT |
| OAXA733 | 1827 | 16.8965 | -96.261935 | CIMMYT |
| GRIN19897 | 1651 | 17.253353 | -96.021444 | GRIN |
| PHP02 | 260 | 42.683049 | -89.019485 | GRIN |
| PHZ51 | 254 | 41.671486 | -93.712203 | GRIN |
| HB229 | 179 | 40.3363 | -89.0022 | GRIN |
| Hickory King | 143 | 38.6270 | -90.1994 | GRIN |
