# Supplementary material for: Aerial root formation in Oaxacan maize (Zea mays) landraces persists into the adult phase and is minimally affected by soil nitrogen and ambient humidity
Source: Front Plant Sci. 2025 Jul 11;16:1607733. doi: 10.3389/fpls.2025.1607733 (PMC12289584; doi:10.3389/fpls.2025.1607733)
Supplement: Supplementary file 9 [file Table2.docx]

Supplementary Table 2. Summary of the experiments conducted in this study, including details on the location, maize genotypes, and measurements, to provide easy access to our research.

| **Experiment** | **Location** | **Genotype** | **Measure parameters** |
| --- | --- | --- | --- |
| Greenhouse study of landraces | Walnut Greenhouse, University of Wisconsin - Madison | PHP02, PHZ51, Hickory King, Oaxa233, Oaxa524, Oaxa733 | -Epicuticular wax  -Days to anthesis  -Number of Nodes with aerial roots |
| 2021 Field experiment - Wisconsin | West Madison Agricultural Research Station, University of Wisconsin - Madison | PHP02, PHZ51, HB8229, Oaxa139, Oaxa524, GRIN19897 | - Epicuticular wax  -Days to anthesis  -Number of nodes with aerial roots (over several weeks) |
| 2022 Field experiment - Georgia | University of Georgia Iron Horse Plant Sciences Farm | Oaxa139, Oaxa524, Oaxa141, Oaxa306, Oaxa612, Oaxa622 | -Epicuticular wax  -Days to anthesis  -Number of Nodes with aerial roots |
| 2022 Field experiment - Wisconsin | Hancock Agricultural Research Station, University of Wisconsin - Madison | PHP02, PHZ51, HB8229, Oaxa139, Oaxa524, Oaxa733, GRIN19897 | -Number of Nodes with aerial roots (three timepoints: early, middle, late season) |
| Greenhouse study of landraces – Three nitrogen levels | Walnut Greenhouse, University of Wisconsin - Madison | GRIN19970, Oaxa139, Oaxa229 | -Number of Nodes with aerial roots  -Stalk diameter  -Average diameter or aerial roots  -Plant height |
| Greenhouse study of landraces – Two humidity levels | Walnut Greenhouse, University of Wisconsin - Madison | PHP02, Oaxa524 | -Number of Nodes with aerial roots  - Number of roots at the top node  -Aerial root diameter |
